# Cutaneous Neuroimmune Interactions in Peripheral Neuropathic Pain States

**DOI:** 10.3389/fimmu.2021.660203

**Published:** 2021-04-12

**Authors:** Daniel B. Lowy, Preet G. S. Makker, Gila Moalem-Taylor

**Affiliations:** School of Medical Sciences, The University of New South Wales, UNSW Sydney, Sydney, NSW, Australia

**Keywords:** neuroimmune, skin, nociceptors, peripheral neuropathy, neuropathic pain

## Abstract

Bidirectional interplay between the peripheral immune and nervous systems plays a crucial role in maintaining homeostasis and responding to noxious stimuli. This crosstalk is facilitated by a variety of cytokines, inflammatory mediators and neuropeptides. Dysregulation of this delicate physiological balance is implicated in the pathological mechanisms of various skin disorders and peripheral neuropathies. The skin is a highly complex biological structure within which peripheral sensory nerve terminals and immune cells colocalise. Herein, we provide an overview of the sensory innervation of the skin and immune cells resident to the skin. We discuss modulation of cutaneous immune response by sensory neurons and their mediators (e.g., nociceptor-derived neuropeptides), and sensory neuron regulation by cutaneous immune cells (e.g., nociceptor sensitization by immune-derived mediators). In particular, we discuss recent findings concerning neuroimmune communication in skin infections, psoriasis, allergic contact dermatitis and atopic dermatitis. We then summarize evidence of neuroimmune mechanisms in the skin in the context of peripheral neuropathic pain states, including chemotherapy-induced peripheral neuropathy, diabetic polyneuropathy, post-herpetic neuralgia, HIV-induced neuropathy, as well as entrapment and traumatic neuropathies. Finally, we highlight the future promise of emerging therapies associated with skin neuroimmune crosstalk in neuropathic pain.

## Introduction

No longer is the skin regarded a purely physical barrier separating an organism from its environment (1). Modern understanding acknowledges the skin as a highly complex protective network against external physical, chemical, and microbiological insult. An intricate network of immune and non-immune cells is responsible for maintaining tissue homeostasis, responding to harmful challenges and promoting tissue repair. Physical structure, antimicrobial biomolecules and immune cell function are among the defensive modalities employed by the skin to protect the host from invasion. Concurrently, cutaneous sensory neurons detect tissue damage and other external stimuli, relaying this information to the central nervous system (CNS).

Far from operating in isolation, tremendous crosstalk occurs between the peripheral sensory nervous system and the immune system. Upon activation, immune cells release a myriad of inflammatory mediators and intercellular messenger chemicals, which can stimulate sensory nerve terminals in the skin. Analogously, nerve terminals can release a cocktail of neuropeptides which modulate immune cell function (2).

While cutaneous neuroimmune interactions are critical in healthy host physiology, their dysregulation is implicated in many disease states, notably psoriasis (3), atopic dermatitis (AD) (4) and allergic contact dermatitis (ACD) (5, 6). It has increasingly become clear that a significant neuroimmune component also contributes to the pathogenesis of neuropathic pain (7). Neuropathic pain refers to pathological pain generated in response to lesion or disease of the somatosensory system (8). Given the varying etiologies and incompletely identified molecular mechanisms resulting in neuropathic pain, investigation into immune system involvement has received much attention (9, 10). Whereas much of this focus has been directed to neuroimmune mechanisms within the damaged nerve, the dorsal root ganglia (DRG) and spinal cord, comparatively little is known regarding the cutaneous involvement in neuropathic pain sensation. Given the vast bidirectional communication between cutaneous sensory nerves and immune cells in various dermatological diseases, it is reasonable to hypothesize a comparable component in neuropathic pain settings. In this review, we discuss cutaneous neuroimmune interactions in physiological and pathophysiological conditions, with particular attention afforded to various peripheral neuropathic pain states.

### Sensory Innervation of the Skin

The skin is predominately innervated by nerves that convey sensory information from the environment to the spinal cord and the brain. These primary afferents are responsible for detecting various sensory inputs including non-harmful stimuli, such as warmth, cooling and light touch, and harmful noxious or painful stimuli. Nerve endings within the skin arise from primary sensory neuron cell bodies in the DRG for body sensations and the trigeminal ganglia for facial sensations (11). Morphologically, these neurons are pseudounipolar; a single axon arises from the cell body before splitting into two branches: one travels toward the skin and the other toward second-order neurons in the CNS.

**Figure 1:**
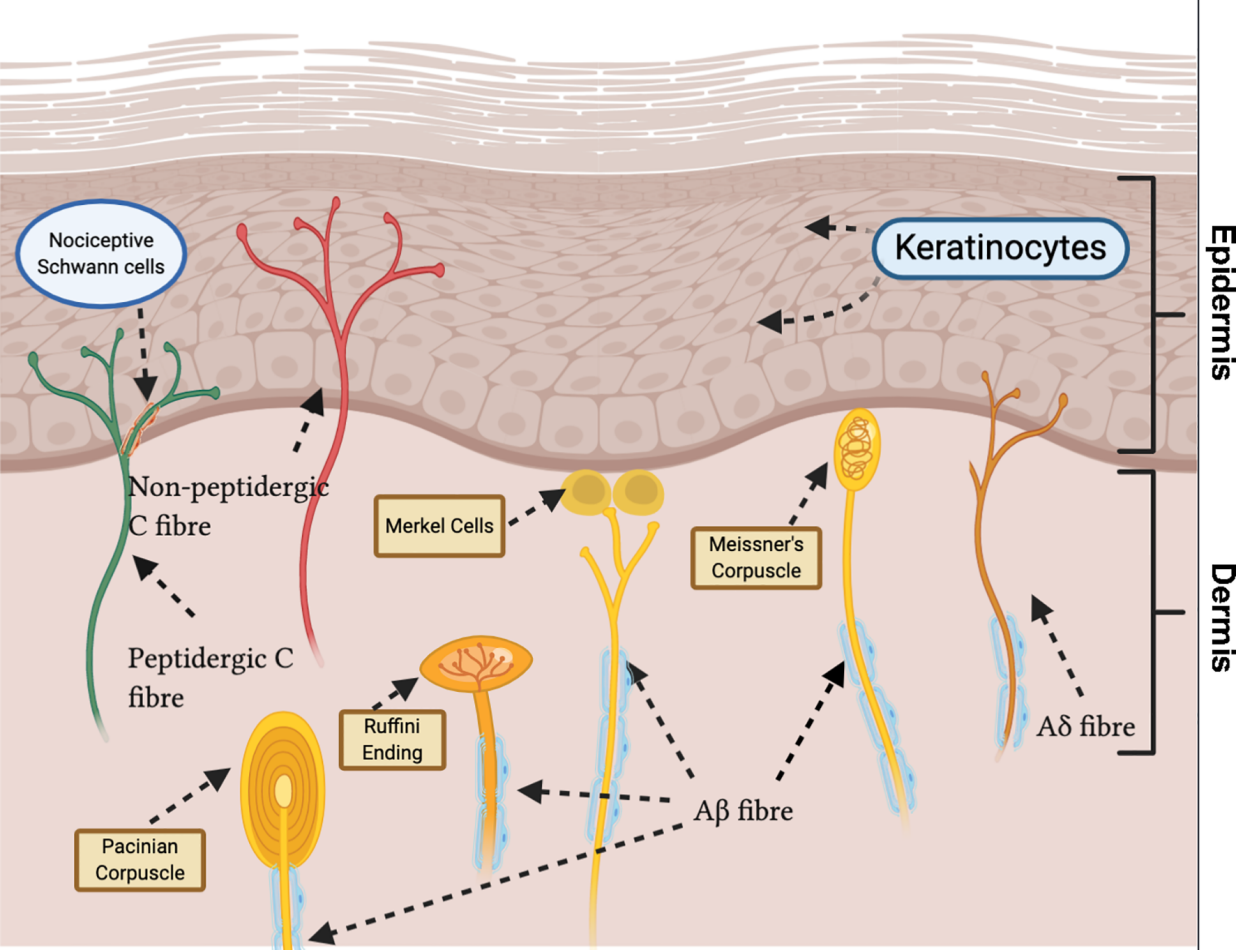
Sensory innervation of glabrous skin displays heterogeneity in nerve ending morphology and end organ type. Unmyelinated C fibres terminate in the deep epidermis (peptidergic) and superficial epidermis (non-peptidergic) as free nerve endings. Aδ fibres terminate in the epidermis as free nerve endings. Recently, novel nociceptive Schwann cells ensheathing free nerve endings have been discovered. Myelinated Aβ fibres terminate as: Pacinian corpuscles, in the deep dermis; Merkel cells, at the epidermal basement; Meissner’s corpuscles, in the dermal papillae; Ruffini endings, in the dermis.

Structurally, the skin can be divided into two main components: the outer epidermis and the deeper dermis. Both layers contain primary sensory afferents, which can be categorized according to degree of myelination, conduction velocity, axonal diameter and nerve ending type (**Figure 1**) (12). These classifications give rise to three types of primary afferents: rapidly-conducting and heavily myelinated Aβ fibers, medium-conducting and moderately myelinated Aδ fibers and slow-conducting, unmyelinated C fibers (13). Axons of the Aβ fibers are of a larger caliber and have a lower threshold of activation compared to Aδ and C fibers, and are mainly associated with detecting light touch, vibration and hair deflection, although Aβ nociceptor (pain) function has been described in mammals (14). Aδ fibers carry information related to temperature and pain. These fibers respond to both mechanical and thermal noxious stimuli. C fibers are the most numerous sensory neurons innervating the skin and are mainly responsible for detecting changes in temperature as well as nociceptive and pruriceptive (itch) stimuli (15). Most C fiber nociceptors are polymodal and can be activated by thermal, mechanical and chemical noxious stimulation (16). However, given the heterogeneity of response to innocuous and nociceptive stimuli across all types, these broad classifications are useful but not exhaustive.

Aβ, Aδ and C fibers can be further distinguished based on their distal terminals within the skin. Aβ fibers generally terminate within the dermis, usually forming close association with hair follicles in hairy skin and various specialized end organs, such as Meissner’s corpuscles, Merkel cells, Ruffini endings and Pacinian corpuscles in glabrous skin. Aδ fibers terminate within the epidermis as free nerve endings or in the dermis of haired skin as circumferential endings (16). Unmyelinated C fibers terminate as free nerve endings in the epidermis between keratinocytes (13). Recently, a novel specialized type of ‘nociceptive Schwann cells’ were observed to envelop free nerve endings. These Schwann cells are located in the epidermal-dermal border, forming a glio-neural end organ, and initiate mechanical pain transduction (17) (**Figure 1**).

C fibers can be broadly classified, neurochemically, into two groups depending on expression of neuropeptides: peptidergic or non-peptidergic (18). Peptidergic neurons express neuropeptides such as substance P (SP) and calcitonin gene-related peptide (CGRP), and terminate in the deep epidermis (19). They also express transient receptor potential vanilloid 1 (TRPV1) (20), a non-selective cation channel, foremost known for its detection of noxious heat (21). Conversely, non-peptidergic C fibers are distinguished by binding isolectin B4 (22) and terminate in the superficial epidermis (19). The majority of non-peptidergic C fibers also express Mas-related G protein-coupled receptor D (MrgprD) (23). However, there is overlap between subsets, with, for example, expression of TRPV1 in sub-populations of non-peptidergic fibers (24). Activation of distinct populations of nociceptors results in diverse pain sensations. Furthermore, after activation, nociceptors can release neuropeptides and other intercellular mediators, modulating immune cell function (20, 25).

### Immune System of the Skin

In the skin, the most superficial layer of the epidermis, the stratum corneum, is the first barrier against hazardous environmental threats. This layer is comprised of flattened keratinocytes that have become denucleated; these cells are known as corneocytes. Lipids fill intercellular spaces between corneocytes to form a physical and chemical barrier, commonly referred to as ‘bricks (corneocytes) and mortar (lipids)’. This hydrophobic layer, which is largely unbroken (in physiological states) except by skin appendages, such as hair follicles and tear ducts, restricts water movement (26). Deep to the stratum corneum is the nucleated epidermal layer, which can be further subdivided into granular, spinous and basal layers.

The most abundant cell type in the epidermis are the keratinocytes, which both produce essential structural proteins and act as immune sentinels to discriminate between innocuous commensal microbes and harmful pathogens (27). Recent studies have demonstrated an additional important role for keratinocytes in mechanical, cold and heat detection *via* keratinocyte-sensory neuron purinergic signalling (28). Toll-like receptors (TLRs) are membrane-bound pattern recognition receptors (PRRs) expressed by keratinocytes and other cell types. TLRs are capable of recognizing molecular patterns frequently found in pathogens (pathogen-associated molecular patterns, PAMPs) and damaged cells (damage-associated molecular patterns, DAMPs) (29, 30). Activation of TLRs can engender inflammatory cytokine and/or chemokine production (31). Additionally, keratinocytes can detect and respond to pathogen infiltration, since keratinocytes are a key synthesizer of antimicrobial peptides (AMPs), especially beta-defensins and cathelicidins (32, 33). AMPs are cationic, amphiphilic biomolecules present across all kingdoms of life. The human body expresses a wide range of AMPs, many of which are expressed in epithelial cells, neutrophils and natural killer cells (34). They mediate first line defense against bacterial, fungal and viral infections (35), although can mediate inflammation if homeostasis is interrupted. In addition to these immune processes, AMPs have been implicated in various intracellular processes, such as wound healing and angiogenesis (36). Further, recent studies have highlighted anti-tumour therapeutic potential; a novel synthetic AMP, Ranatuerin-2PLx, inhibited prostate cancer cell proliferation *in vitro* (37). The role of keratinocytes in patients with small fiber pathology has recently been studied using transcriptomic analysis of skin biopsies, revealing an enrichment of inflammatory pathways with higher expression of algesic mediators in keratinocytes (38).

**Figure 2:**
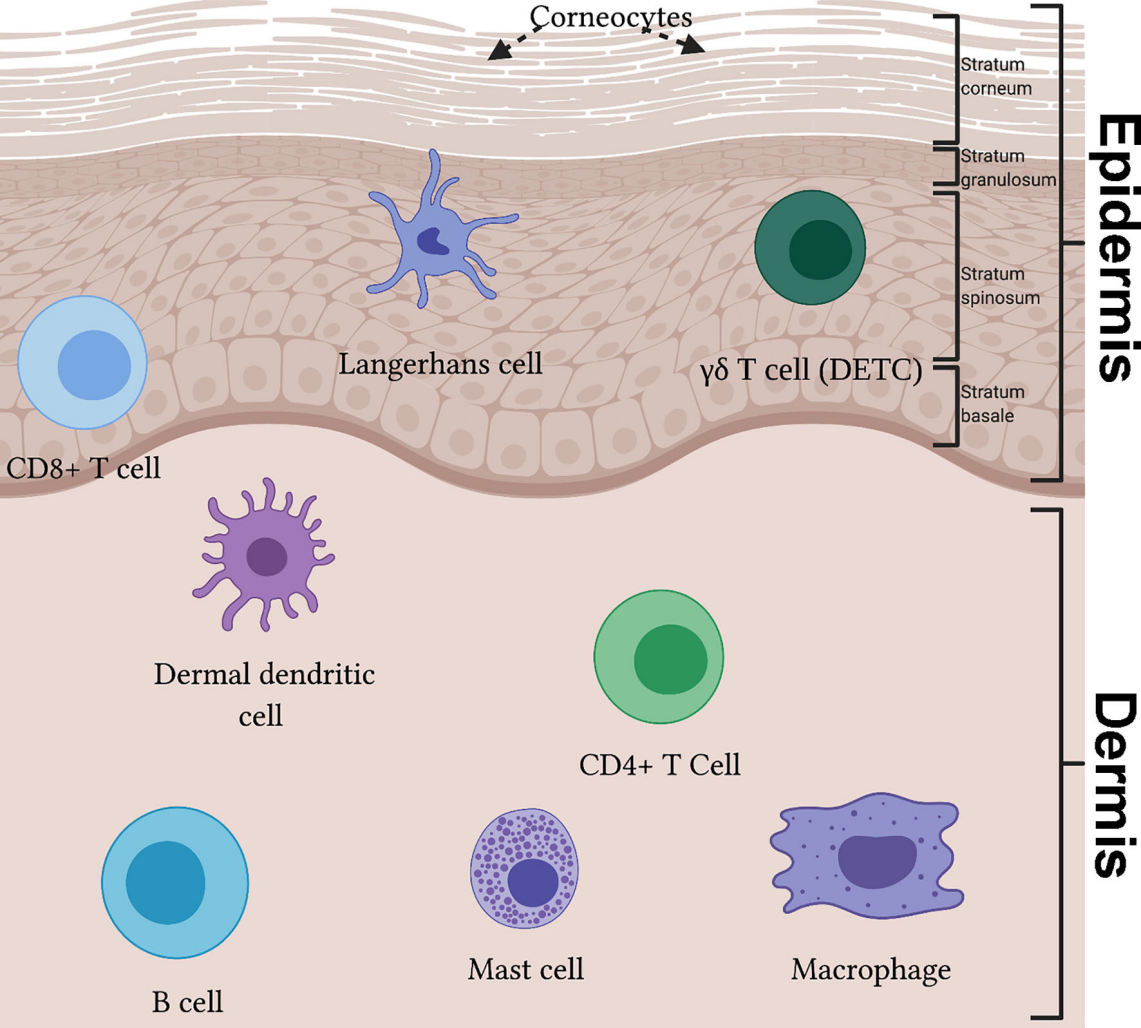
The immune system of the skin reveals a diverse population in effector immunocytes. The epidermis can be divided into four layers, from superficial to deep: stratum corneum, stratum granulosum, stratum spinosum and stratum basale. In the epidermis, Langerhans cells partake in antigen presentation and migration to lymph nodes. γδ T cells, also known as dendritic epidermal T cells (DETCs), are key cytokine producing cells in mice skin. Cytotoxic CD8+ T cells can be found in the deep epidermis. Within the dermis resides many specialized immune cell populations, including dermal dendritic cells, CD4+ T cell subsets (e.g. Th1, Th2, Th17 and T follicular helper) and B cells. Additionally, mast cells and macrophages are present.

Skin-resident immune cells play a key role in skin immunosurveillance, functioning as sentinels for the innate and adaptive immune system by recognizing foreign antigens. Major players in the skin’s immune system include myeloid cells, such as Langerhans cells (LCs), dermal dendritic cells (dDCs), macrophages, and mast cells, in addition to lymphocyte cells such as T cells and B cells (**Figure 2**) (39). These cells are responsible for both mounting immune responses when the host is threatened and for maintaining normal physiology in homeostatic conditions.

Langerhans cells are the sole resident myeloid cell of the epidermis, contributing to the physical and immunological skin barrier. LCs have a dendritic phenotype; their projections propagate through keratinocyte tight junctions in the epidermis, allowing them to detect antigens throughout this layer without disrupting its permeability (40). These cells are key antigen-presenting cells of the skin and are able to migrate to the lymph node enabling dual-function: induction of tolerance during homeostasis (41) and the presentation of antigen to elicit an adaptive immune response in an inflammatory setting (42).

Dermal dendritic cells reside in the dermal layer and, similar to LCs, traffic foreign antigens to lymph nodes to induce adaptive immune response during pathogen invasion (43). In fact, the presence of external pathogen in concert with inflammatory signalling drives functional maturation of dDCs (44). In lymphoid organs, dDCs are highly significant modulators of T-cell activation, mediating either tolerance or sensitization of T-cell response. In the former framework, resting dDCs can induce antigen-specific tolerance in CD8+ T-cells (45). In other words, steady-state dDC function can inhibit T-cell activation thereby reducing potential autoimmune interactions. Conversely, antigen cross-presentation by dDCs, and other antigen-presenting cells, to CD8+ or CD4+ T-cells elicits an antigen-specific immune response. Hence, such sensitization results in clonal expansion and acquisition of appropriate cytotoxic function specific to the invading pathogen (44).

Macrophages are myeloid-derived mononuclear phagocytes which are known for their canonical roles in innate immunity and phagocytic function (46), wound healing (47), tissue repair (48), hair follicle development (49) and basal cell carcinoma prevention (50). They are the predominant innate immune cell found within skin, with recent evidence identifying a subset of dermal macrophages contributing to sensory nerve surveillance and repair (51). Further, distinct profiles of the heterogenous dermal macrophage population were established, with a subset of specialised macrophages associated with sensory nerve axon patrol (51). In homeostatic state, macrophages are found in the dermis and partake in the clearance of cellular debris and neutrophil recruitment *via* cytokine release (39).

Mast cells are pleotropic bone marrow-derived cells which play a fundamental role in immunity, inflammatory and allergic reactions and are typically located at host-environment junctions such as the skin dermis (52). Their diverse range of skin effector functions include mediating hypersensitivity, allergic response, and parasite elimination (53). Once activated, mast cells release a variety of pre-formed (e.g. histamine, proteoglycans, serotonin) and *de novo* (cytokines, chemokines, growth factors) synthesized mediators (54, 55). Mast cells participate as first-line responders of the immune response, instigating bidirectional communication with other immune cells.

T cells are lymphoid cells that differentiate and develop in the thymus before becoming key cells in responding to tissue insult and maintaining host homeostatic architecture. Adult human skin contains approximately 20 billion T cells (56). In human skin the αβ T cell is the predominant T cell type, based on T cell receptor dimer (39). γδ T cells are the predominant T cell type in murine skin (57). A recent identified subpopulation of double-positive αβγδ T cells in human fetal skin has been described with potential role in skin development and protection of the fetus against intrauterine infections (58).

The skin contains a proportion of both resident (T_RM_) and recirculating T cells (59). CD4+ T_RM_ are the most prevalent T_RM_ in the skin and are distributed across the epidermis and dermis (60). Upon specific antigen interaction, a naïve CD4+ T cell can differentiate into one of many effector subgroups, including conventional CD4+ helper T (T_h_) (e.g. Th1, Th2, Th17 and T follicular helper) and regulatory T (T_reg_) cells (61). T_h_ cells assist in combating intracellular pathogens, extracellular pathogens, clearance of extracellular microorganisms at mucosal surfaces, modulating B cell response and suppressing pathogenic immune response mediated by self-reactive effector cells (61, 62). T_reg_ cells facilitate hair follicle regeneration, promote wound healing and protect against commensal microbial infection (62). CD8+ T_RM_ are instrumental in the cytotoxic clearance of intracellular pathogens, autoimmunity, allergic response, anti-tumour response and immunity to viral infection (63). They are usually found in the epidermis and are implicated in a number of pathological states such as psoriasis (3), vitiligo (64) and allergic dermatitis (6). Most γδ T cells in murine skin are known as dendritic epidermal T cells (DETCs) as they project dendritic processes throughout the epidermis. Activated DETCs retract their dendrites and are key producers of interleukin (IL)-17, an essential inducer of beta-defensins in the epidermis (39) and a key signaler in cutaneous diseases like psoriasis (65).

Until recently, it was thought that B cells had a negligible role as resident cells in healthy skin. Recent data have suggested localization of B cells during homeostasis in both murine and human skin (66, 67). In addition to their canonical role as instigators of humoral immunity, B cell antibody secretion *via* eccrine sweat glands has been implicated in the regulation of microbial environments on the skin surface (68). Further, B cells localize to skin lesions and may contribute to wound healing (69, 70). The role of B cells during skin inflammatory responses is better characterized. B cells are key drivers of inflammation across various disorders, including melanoma (71), AD (72), and autoimmune diseases such as pemphigus vulgaris (73, 74). Resultantly, B cell-targeted depletion therapies for several dermatological disorders have garnered successful results (74). There is evidence to suggest a population of B cells which have the capacity to dampen inflammation and autoimmune response, coined regulatory B cells (B_regs_) (75). B_regs_ secrete IL-10, an anti-inflammatory cytokine which limits pro-inflammatory cytokine production in T cells and antigen presentation function in dendritic cells (76). Ultimately, B cell function can be both stimulating and regulatory of immune response through provision of variable cytokines.

## Immune Cell Regulation by Sensory Neurons in the Skin

In addition to their primary role of transmitting signals from the periphery to the spinal cord and brain, somatosensory neurons mediate immune responses through the release of neuropeptides and neurotransmitters from peripheral terminals. Innate and adaptive immunocytes express receptors for sensory neuron-derived factors, facilitating direct communication between nervous and immune cells. Sensory neurons have been shown to modulate immune response in models of infection and inflammation (77–79). For example, recent findings in a mouse model of cutaneous allergen exposure demonstrate that allergen-induced SP release by TRPV1+ sensory neurons stimulates dendritic cell migration to the lymph node, where they initiate Th2 cell differentiation and allergic immune response (80). Similarly, recent evidence points to a role of lung nociceptor neurons in initiating downstream Th2 cell activation and subsequent allergen-induced inflammation (81). Another recent study using optogenetic activation of nociceptors in naïve mice demonstrated that levels of dDCs and γδ, αβ T cells and DETCs increased in the skin after optogenetic stimulation (82). Further, optogenetic nociceptor stimulation in mice treated with complete Freund’s adjuvant induced significant additional increases in the numbers of γδ and αβ T cells in the inflamed skin indicating that antidromic nociceptor activation alone is sufficient to induce and potentiate inflammation (82). This study further illustrates the strong links between sensory neurons and immune cells in the skin.

Modulation of immune response by sensory neurons has been implicated in skin infections and in several inflammatory skin conditions, including ACD, AD and psoriasis.

Microbial skin infections can be associated with pain. Evocation of pain has a protective function, eliciting defensive actions in response to harmful stimuli. Traditionally, local inflammatory response involving the recruitment of immune cells was thought to drive bacterial infection-induced pain. Immune cells release mediators that sensitize peripheral sensory neurons *via* specific receptors on neuronal surfaces. Activation of these receptors modulates ion channel activity and enhances action potential generation, resulting in pain sensation (83). However, it was shown that pain elicitation during *Staphylococcus aureus* infection is not immune-mediated but rather due to direct activation of nociceptors by bacterial-derived mediators (84). Further, depletion of voltage-gated sodium channel 1.8 (Nav1.8) positive neurons was associated with nullification of mechanical and thermal hyperalgesia (exaggerated response to noxious stimuli) in addition to increased neutrophil and macrophage infiltration to the site of infection (84). Baral and colleagues also found that Nav1.8+ neurons were involved in bacterial dissemination from lungs to extrapulmonary sites (85). These data implicate an involvement of Nav1.8+ nociceptors in both pain transduction and modulation of the local inflammatory response following bacterial infection. Similar mechanisms of pain transduction and immune regulation have been illustrated in mice models of *Streptococcus pyogenes* skin infection (79). This study demonstrated that pain during infection was mediated by bacterial release of pore-forming toxin, streptolysin S (SLS), which triggers release of CGRP by sensory neurons. Administration of botulinum toxin-A (BTX-A), an inhibitor of neuronal vesicular release, or CGRP receptor antagonist were associated with increased bacterial clearance in the skin and heightened local neutrophil recruitment (79). In a murine model of cutaneous *Candida albicans* infection, nociceptors directly sensed the fungal pathogen and released CGRP, driving the production of pro-inflammatory cytokine IL-23 in CD301+ dDCs, which in turn induced IL-17A production from γδ T cells, inhibiting infection (86). These studies indicate pathogenic involvement in the release of sensory neuron-derived neuropeptides leading to immune response modulation.

ACD is a widely prevalent skin condition caused by a type IV hypersensitivity reaction initiated by skin contact with allergens resulting in interaction with antigen-specific T cells and peptidergic nerve fibers (87, 88). Inflammatory response to allergen (haptens, oxazolone and urushiol) challenge was diminished during both pharmacological blockade of the nociceptor channel transient receptor potential ankyrin 1 (TRPA1) and in *Trpa1*-knockout mice (89). Further, skin biopsies of allergen-treated TRPA1 knockout mice revealed diminished levels of pro-inflammatory cytokines and endogenous pruritogens, including SP (89). Squaric acid dibutylester is a hapten used to induce a mouse model of ACD and was shown to directly activates both TRPV1 and TRPA1 channels, mediating persistent itch. Further, pharmacological or genetic ablation of TRPV1 channels resulted in macrophage-mediated inflammatory edema (90). Both studies indicate a potential neural regulatory component in ACD pathophysiology.

AD, or eczema, is a prevalent chronic or recurrent inflammatory skin condition characterized by acute ‘flare-ups’ of pruritic lesions over dry skin (91). Chronic itch is a hallmark feature of AD, mediated by the crosstalk between non-histaminergic sensory fibers, keratinocytes and immune cells (4). A recent study has demonstrated that while mast cells are required for acute allergic itch, a unique subset of IgE receptor+ basophils localize near free nerve endings and drive allergen-evoked itch flare ups in AD (92). During such a flare, degranulation of basophils releases leukotriene C4 causing activation of sensory neurons *via* its receptor CysLTR2, initiating itch signalling (92, 93). Intraepidermal nerve fiber (IENF) density is higher in the skin of human AD patients compared to healthy controls (94). Similar findings were observed in a mouse model of AD (95). An *in vitro* skin model of AD demonstrated enhanced neurite outgrowth, increased epidermal innervation and proliferation of keratinocytes, the latter observation driven by neuronally-derived CGRP (96). A small study of AD patients found an increase in the number of SP+ and neurokinin A+ nerve fibers in lesioned skin. Interestingly, topically administered SP was shown to restore normal epidermal barrier function and reduce epidermal nerve infiltration in a NC/Nga mouse model, representative of human AD (97). Recently, Serhan et al. (98) demonstrated the initiation of allergic response, a key early event of AD, through the activation of SP-producing TRPV1+ nociceptors. Once activated, this neuronal population releases SP which then triggers mast cell degranulation *via* the mast cell receptor MRGPRB2 (98, 99). These studies indicate that effector functions of peripheral sensory neurons may be key drivers of AD development.

Psoriasis is a complex inflammatory skin condition whose pathogenesis is not completely understood. To many, psoriasis is understood as a T cell-mediated autoimmune disease, underscored by both dysregulation in the T cell-orchestrated IL-17/IL-23 axis (65) evident during the disease process and effectiveness of targeted T cell therapies (3). However, a raft of observations lend support to the hypothesis of neural involvement in the maintenance of psoriasis. These include the symmetry of plaque distribution (100), the proliferation of cutaneous sensory fibers and of sensory neuron-derived neuropeptides in psoriatic skin (101) and the inhibition of spontaneous behaviors in an imiquimod-induced mouse model of psoriasis due to sensory denervation and the blockade of sensory neural mechanisms (102). Elmariah et al. (103) reviewed cases of alterations in manifestation of pre-existing inflammatory skin disease (including psoriasis and AD) in patients with acquired neurological damage. Resolution of skin lesions occurred in regions innervated by injured nervous tissue in 19 of 23 cases. Moreover, in one patient psoriatic lesions reappeared 4 months after nerve injury, coinciding with recovery of affected nerve supply (104). Additionally, axotomy of cutaneous nerves in a KC-Tie2 psoriasiform model resulted in improved acanthosis and reduced CD4+ T cell and CD11c+ dDC numbers (77). Further, it was shown that TRPV1+/Na_v_1.8+ sensory neurons regulate the IL-17/IL-23 axis by interacting with dDCs (78). Ablation of this neuronal subset attenuated dDC production of IL-23 and subsequently reduced IL-23-dependent IL-17 release by γδ T cells, diminishing inflammatory response and thus underlining the regulatory role exercised by cutaneous sensory nerves in psoriasis (78). Moreover, optogenetic activation of cutaneous TRPV1+ fibers was shown to be sufficient in initiating a CGRP-dependent psoriasis-like IL-17 local immune response (105). In addition, nerve activation triggered a nerve reflex arc resulting in anticipatory immune response in adjacent unstimulated skin (105).

## Sensory Neuron Regulation by Immune Cells in the Skin

Numerous studies have shown that immune cells and their mediators in the skin can activate peripheral nerve terminals. Tissue injury is usually associated with an inflammatory response that is coupled to pain sensitization. Nociceptor terminals can detect inflammatory mediators that are released by immune cells, resulting in nociceptive signalling (83). Acute nociceptive pain usually resolves with the resolution of local immune response, although chronic immune signalling can instigate chronic pain states.

Mast cells are understood to regulate inflammation-associated pain, facilitated by their close anatomical proximity to peripheral nerve terminals (106). Depending on activating stimulus, mast cells can secrete a wide range of mediators including cytokines such as IL-1ß, IL-5, IL-6 and tumour necrosis factor (TNF), tryptase, histamine, serotonin, and nerve growth factor (NGF), of which all are capable of binding to specific receptors on nociceptor terminals (107, 108). This results in nociceptor sensitization and contributes to pain during both acute and chronic inflammation (107, 109). Mast cell degranulation also regulates itch sensation *via* histaminergic and non-histaminergic pathways. Histaminergic itch is defined by immunoglobulin E (IgE)-mediated degranulation of mast cells and subsequent histamine release (5). Recent evidence has recognized non-histaminergic activity of mast cells, characterized by Mas-related gene X2 receptor (MrgprX2) activation, degranulation of chiefly tryptase rather than histamine and activation of distinct pruriceptors (110). In all, mast cells are responsible for activating peripheral sensory nerve fibers which in turn secrete mast cell-activating neuropeptides, facilitating bidirectional signalling.

Neutrophils are the first recruited innate immune cell in response to tissue injury or insult and are responsible for the clearance of pathogens and cellular debris. Evidence of direct cutaneous neuroimmune crosstalk orchestrated by neutrophils is limited. However, migratory neutrophils release inflammatory mediators such as prostaglandin E_2_ (PGE_2_), TNF and interleukins at the site of injury, contributing to nociceptor sensitization (111, 112). Recent evidence has demonstrated that infiltrating neutrophils are essential instigators of itch in a mouse model of AD (113), as they upregulate the itch-inducing chemokine C-X-C motif chemokine 10 (CXCL10) which drives itch *via* activation of its receptor, CXCR3, on sensory neurons (114).

Peripheral macrophages modulate initiation, maintenance, and resolution of pain signals by directly interacting with peripheral nociceptors. Macrophages release pro-inflammatory mediators such as TNF, IL-1β and PGE_2_ which act on their respective receptors expressed by nociceptors leading to peripheral sensitization (25). Skin macrophages are critical in the development of Angiotensin II (AT2)- induced nociceptor sensitization (115). Activation of type 2 AT2 receptor, expressed by peripheral macrophages and not expressed by human and mouse sensory neurons, results in the production of reactive oxygen/nitrogen species which activate nociceptors in a TRPA1-dependent manner (115). This interaction may represent a peripheral mechanism of chronic/neuropathic pain signalling and therefore provides a potential pharmacological target. Further, skin macrophages are key participants in both thermal and mechanical hyperalgesia mediated by the complement system (116, 117), a key player of the innate immune system characterized by its contribution to inflammatory and neuropathic pain (118, 119). Complement system component C5a induces macrophage-dependent thermal and mechanical sensitization, involving the release of NGF and CGRP, respectively (116, 117). In both studies, the macrophage-derived neuropeptides activated TRPV1+ nociceptors, eliciting hyperalgesia (116, 117). TRPV4 is implicated in both acute and chronic itch. Luo et al. (120) showed that TRPV4 is selectively expressed by macrophages and keratinocytes in murine skin. Further, the study demonstrated that activation of these channels results in itch-specific behaviors, *via* down-stream serotonin activation of 5-hydroxytryptamine (5-HT) receptors located on itch-sensing fibers. Recent evidence has suggested that macrophages play a role in resolution of pain by adopting an anti-inflammatory phenotype. Macrophages express GPR37, a receptor of neuroprotectin D1, a specialized pro-resolving mediator involved in inflammation resolution (121). Activation of GPR37 by neuroprotectin D1 results in a switch from pro- to anti- inflammatory signalling *via* increased release of IL-10 and transforming growth factor β (TGF-β) and attenuated release of IL-1β and TNF (121). Established and recent evidence support diverse phenotypes of macrophages in a neuroimmune context.

Evidence supporting T cell regulation of cutaneous sensory nerves is limited. The cytokine IL-31 is implicated in pruritic skin conditions, such as AD and cutaneous T cell lymphoma (122, 123). IL-31 is typically derived from Th2 cells and was shown to activate IL-31 receptor alpha (IL-31RA) expressed by TRPV1+/TRPA1+ cutaneous nerves, mediating itch (124). Further, IL-31 induced sensory nerve elongation and branching, resulting in increased nerve fiber density, both *in vitro* and *in vivo* (125).

## Skin Neuroimmune Interactions in Peripheral Neuropathy

Whereas acute nociceptive pain warns and protects an organism in response to injury/inflammation, chronic neuropathic pain may persist beyond usefulness and arises due to maladaptation of the somatosensory nervous system. Chronic neuropathic pain can present with symptoms of pain hypersensitivity and paraesthesia/dysesthesia (perception of abnormal sensation, such as tingling and pins and needles) (126). Resolution of chronic neuropathic pain is limited by moderate efficacy of available treatments and associated severe adverse effect profiles, underscoring the need to elucidate the molecular mechanisms underpinning neuropathic pain as a prerequisite of novel drug development (127). The molecular mechanisms that contribute to peripheral nociceptor hyperexcitation in neuropathic pain have been widely studied utilizing several animal models, typically rodent models of traumatic peripheral nerve injury, toxic and drug-induced neuropathy, and models involving the induction of a disease state which mimics a specific clinical neuropathic pain state (127).

These models have demonstrated that neuropathic pain is characterized by dysregulation of the pro-/anti-inflammatory balance resulting in excessive neuroinflammation at both the site of nerve injury and at the sensory nerve terminals. In this setting, sensitized nociceptor terminals can undergo deleterious plastic transformation, characterized by altered expression of receptors, channels, transporters and neurotransmitters (128). A variety of etiologies can cause peripheral nerve damage and neuropathic pain. Common causes include metabolic dysfunction [e.g. diabetic neuropathy (129)], drug and toxin exposure [e.g. following treatment with neurotoxic chemotherapy (130)], trauma and infection [e.g. post-herpetic neuralgia (131)]. The contribution of cutaneous neuroimmune interactions to neuropathic pain caused by chemotherapy-induced peripheral neuropathy (CIPN), diabetic neuropathy, viral disease-induced neuropathy, and entrapment and traumatic neuropathies are discussed below. This list is not exhaustive, and other neuropathic pain conditions such as autoimmune neuropathies are beyond the scope of this review.

### Chemotherapy-Induced Peripheral Neuropathy

CIPN is a common, dose-limiting side-effect of chemotherapy use (132, 133) and is associated with a range of chemotherapeutic drug classes i.e. vinca alkaloids, platinum compounds, taxanes, thalidomide derivatives and proteasome inhibitors (134). CIPN most commonly manifests as a sensory or a mixed sensorimotor axonal neuropathy with symptoms of paraesthesia, dysesthesia and neuropathic pain that often present in a stocking-glove distribution (133, 135). Current treatments of CIPN are largely inefficacious and presently CIPN is considered an unavoidable complication of chemotherapeutic use (136). The mechanisms of CIPN and associated neuropathic pain are varied and involve modulation of ion channels in sensory neurons causing altered peripheral nerve excitability (137–140), and neuroinflammation characterized by infiltration of immune cells, activation of cytokines and changes in glial cell activity in the peripheral sensory nerve apparatus (130, 141–143). However, the important role of cutaneous neuroimmune interactions involving distal nerve fiber endings in chemotherapy-induced neuropathic pain is emerging.

**Figure 3:**
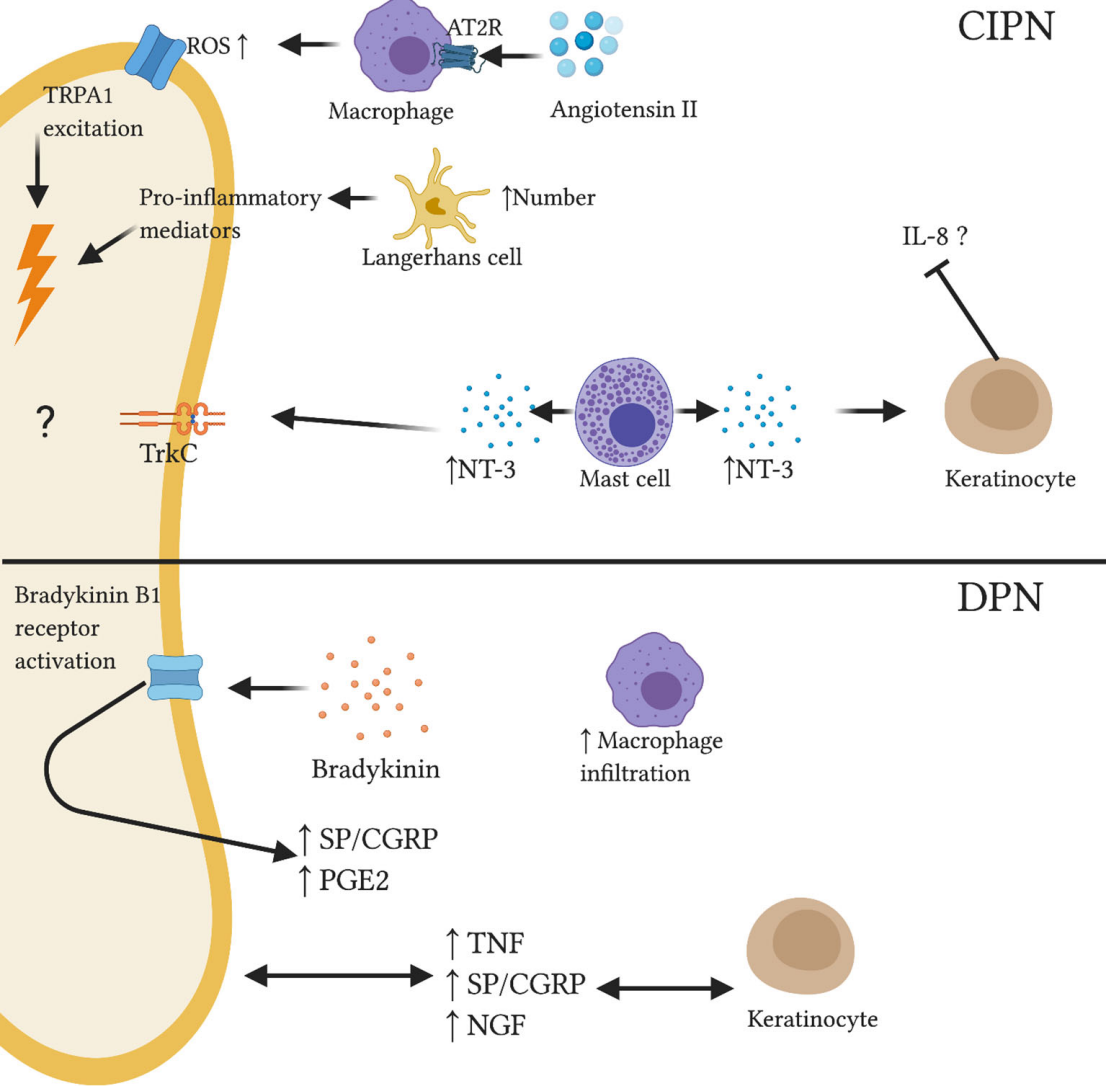
Reported and speculative neuroimmune interactions within the skin in CIPN and DPN. In a CIPN mouse model, Angiotensin II administration resulted in macrophage release of reactive oxygen species via AT2R. TRPA1 stimulation caused nociceptor excitation. Increased number of activated Langerhans cells may release pro-inflammatory mediators which can sensitize nociceptors. Altered expression of neurotrophins such as NT-3 may affect immune and neuronal function; elevated levels of mast cell-derived NT-3 associated with reduced IL-8 production from keratinocytes in skin of AD patients. In DPN, abnormal neurotrophin signalling may disrupt keratinocyte and neuronal function. Bradykinin administration was shown to upregulate inflammatory neuropeptides SP and CGRP and inflammatory lipid PGE2. Further, increased macrophage infiltration was observed in the skin of diabetic mice.

Numerous studies in rodents and skin biopsy of CIPN patients have demonstrated loss of IENFs indicating IENF degeneration due to chemotherapy-induced neurotoxicity (144–148). In addition, pre-clinical and human studies have indicated the role of cutaneous neuroimmune interplay in the pathogenesis of CIPN. Shepherd et al. (115) showed activation of type 2 AT2 receptor on skin macrophages resulted in reactive oxygen species-dependent excitation of TRPA1 channels on sensory nerves causing mechanical hypersensitivity (**Figure 3**). AT2 intradermal injection induced macrophage infiltration in the mouse hindpaw skin and increased macrophage density in skin biopsies of CIPN and diabetic patients (115). Additionally, murine models of paclitaxel- and vincristine-evoked painful neuropathies have shown significant reduction in hindpaw IENF number and an increase in activated LCs (146). There is evidence to suggest that activated LCs release amplified amounts of pro-inflammatory cytokines which can sensitize nearby intact C-fibers (149). A recent longitudinal cohort study assessed the therapeutic potential of topical high-concentration capsaicin 8% patch application in chronic CIPN patients no longer receiving chemotherapy; capsaicin 8% patch provided significant reduction of CIPN-related pain (150). Capsaicin is rapidly released from the patch, activating TRPV1 channels and overstimulating nociceptors. This results in a short-term degeneration of these sensory fibers before the induction of regeneration and restoration of a healthy nerve fiber phenotype (151). Baseline skin biopsies of CIPN patients revealed lowered NGF, significantly increased neurotrophin-3 (NT-3) and slightly increased LC number compared to controls (150). Decreased NGF expression in CIPN patients may occur due to the toxicity of chemotherapy toward keratinocytes, which secrete NGF (152). NT-3 is, along with NGF, a member of the neurotrophin family and plays an important role in neuronal growth and regeneration (153). Increased cutaneous expression of mast cell-derived NT-3 was shown in skin biopsies of patients with AD causing reduced IL-8 production from keratinocytes (**Figure 3**) (154). A similar mechanism may occur in CIPN. Following capsaicin patch treatment, epidermal levels of NGF, NT-3 and LCs approached healthy levels. A recent study demonstrated a relationship between paclitaxel-induced degenerative loss of SP+ and CGRP+ peptidergic IENF density and the development of neuropathic pain in a rat model (145). The mechanisms underpinning this observation are not completely understood; it could be speculated that altered SP and CGRP release from degenerating fibers may influence the cutaneous microenvironment. Interestingly, the correlation between decreasing peptidergic IENF density and development of neuropathic pain behaviors is not ubiquitous across neuropathic pain models.

### Diabetic Polyneuropathy

Diabetes is set to reach 366 million affected individuals by 2030, reaching pandemic levels (155). Diabetic polyneuropathy (DPN) is a common microvascular complication of diabetes and approximately 20-30% of patients with DPN experience neuropathic pain (156). Sensory dysfunction associated with DPN can manifest as a range of sensory symptoms including both hyperalgesia and hypoalgesia, associated with short-term and long-term diabetes, respectively (157, 158). The most common murine model used to study DPN is streptozotocin- (STZ) or alloxan-induced which are toxic to the pancreatic insulin-secreting cells (159).

The peripheral pathophysiology of DPN has not been clearly elucidated. Increased levels of pro-inflammatory mediator TNF were seen in the skin of STZ-induced mice (160). Footpad inoculation of herpes simplex virus (HSV)-based vector expressing p55TNF soluble receptor inhibits TNF pro-inflammatory signalling (161). Subcutaneous injection of this vector attenuated diabetes-induced hyperalgesia and restored normal TNF mRNA levels in the skin (160). Numerous rodent models of DPN have reported progressive declines in IENF density (162–164). In contrast, a recent study showed increased density of dermal peptidergic (SP+/CGRP+) fibers in human patients with painful DPN compared to painless DPN and healthy controls (165). Thus, dermal peptidergic nociceptor density may serve as a cutaneous marker for painful DPN. A preclinical study employing the STZ rat model of DPN also showed significantly increased trkA+/CGRP+ IENF and significantly elevated NGF levels in the skin compared to controls (166). These data indicate that elevated skin levels of NGF may result in over-expression of CGRP-labeled nociceptors and contribute to neuropathic pain behaviors. However, skin isolated from another STZ diabetic study found lower basal levels of SP and CGRP compared to healthy controls while PGE2 was similar between groups (167). Bradykinin treatment evoked increased SP, CGRP and PGE2 release from peripheral nociceptors across treatment and control groups. Although relative change from basal levels was significantly higher in diabetic skin, peak levels were similar across both groups (167). This observation indicates functional involvement of neuronal bradykinin B1 receptors- constitutively expressed at low levels but upregulated in chronic inflammatory states- in stimulating excessive neuropeptide release thereby contributing to diabetic hyperalgesia (**Figure 3**). Indeed, this hypothesis is supported by growing evidence of the pathological neuroimmune processes mediated by bradykinin signalling in cutaneous healing and disease states such as psoriasis (168, 169). Recently, a novel whole-mount imaging method of murine ear skin enabled visualization of abnormal peripheral nerve morphogenesis in both diet-induced obesity and leptin receptor-deficient *db/db* models of type 2 diabetes (170). Further, increased macrophage infiltration was seen in the skin of diet-induced obesity mice compared to controls, but not in *db/db* mice skin (170). Accordingly, the authors suggested that inflammatory immune cell infiltration may not be a primary driving force behind the observed neuronal abnormalities in type 2 diabetes (170). Though the extent to which inflammatory mediators and immune cells contribute to cutaneous pathology in DPN is unclear, emerging clinical and preclinical evidence warrants further research in this area.

### Viral Disease-Induced Neuropathy

Post-herpetic neuralgia (PHN) is a chronic condition characterized by neuropathic pain persisting for 3 months or more after an outbreak of shingles (171). Shingles, also known as acute herpes zoster (AHZ), occurs due to the reactivation of dormant varicella zoster virus (VZV), the same virus causing chicken pox in children. Following initial VZV infection, the virus remains dormant in sensory ganglia and can reactivate later in life, resulting in AHZ. Approximately 10% of AHZ patients develop PHN, establishing the condition as the most common infectious etiology of neuropathic pain (172).

As VSV-specific cell-mediated immunity falls, anterograde transport (from ganglia to peripheral terminals) of the dormant virus to the affected cutaneous dermatome triggers an array of cellular responses in both infected and non-infected resident skin cells. Skin biopsies of affected dermatomes reveal a reduction in cutaneous sensory afferent innervation (173). Loss of cutaneous innervation parallels severity of neuropathic pain (174). This reduction causes a decrease in neuropeptide (CGRP, SP and vasoactive intestinal polypeptide [VIP]) release, dysregulating local cutaneous immunity (175, 176). As a result, VSV-affected dermatomes become predisposed to the subsequent development of immune-mediated disorders, including malignant tumors, opportunistic infection and autoimmune disease (177). The phenomenon is characterized as “post-herpetic isotopic response”. Examination of neuropeptide signalling in PHN-affected dermatomes may reveal changes in the cutaneous neuroimmune environment.

**Figure 4:**
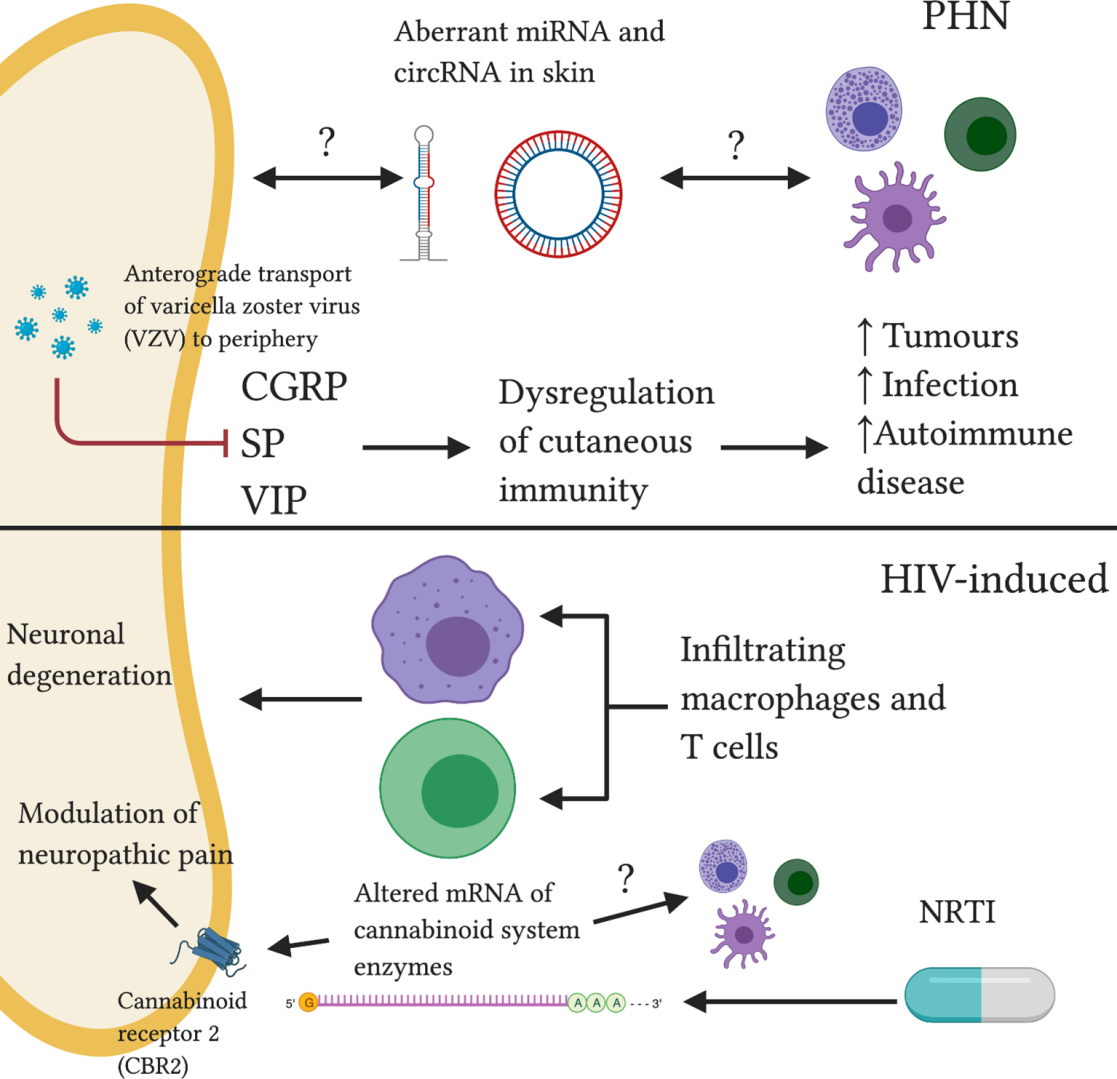
Reported and speculative neuroimmune interactions within the skin in PHN and HIV-induced neuropathy. In PHN, anterograde transport of VSV results in dysregulation of cutaneous neuropeptide (CGRP, SP, VIP) signalling, increasing susceptibility to various pathologies. Altered ncRNA balance may modulate neuronal and immune function. HIV-induced neuropathy is associated with infiltrating neurotoxic macrophages and T cells. NRTI treatment drives abnormal cannabinoid system signalling, a system implicated in the pathogenesis of neuropathic pain.

Peripheral mechanisms of PHN are not fully elucidated and are subject to debate (178). MicroRNA (miRNA) and circular RNA (circRNA) are non-coding RNA (ncRNA) molecules which can modulate gene expression (179, 180). Their aberrant expression has been implicated in several disease states (181). Differential profiles of miRNA and circRNA expression in PHN skin compared to unaffected skin has been reported (**Figure 4**) (182). Whether the differential miRNAs and circRNAs were located within nerve endings, skin immune cells or extracellular space is unclear. Importantly, recent mounting literature has suggested that ncRNAs may act as ‘master switches’ in regulating the neuroimmune balance within the skin (183, 184). Further investigation into ncRNA function may present exciting diagnostic and treatment applications for neuropathic pain states.

Antiretroviral therapy has converted human immunodeficiency virus (HIV) infection from a progressive, fatal illness to a manageable chronic condition (185). Neuropathic pain is a major source of morbidity among HIV-infected/AIDS patients with a prevalence of 20-40% (186, 187). HIV-induced neuropathy and associated neuropathic pain occurs due to neurotoxic effects of the virus and also the neurotoxicity of front-line antiretroviral drugs (185).

A hallmark of HIV-induced sensory neuropathy is a loss of IENF density (188) although few studies have investigated immune contribution to this phenomenon. Mountford et al. (189) found that infiltrating macrophages and T cells expressing chemokine receptors CX3CR1 and CCR2/CCR5, respectively, in the skin of HIV patients may contribute to cutaneous nerve fibre degeneration. In an earlier study, CX3CR1 knockout mice demonstrated attenuation in inflammatory and neuropathic pain responses, indicating a role for the chemokine receptor in pain transduction (190). Similarly, CCR2 has been implicated in pain behavior following nerve injury (191) and CCR5 is understood to serve as a receptor for HIV-1 envelope glycoprotein 120 (gp120) on nerve cells, facilitating direct neuronal damage (192).

Nucleoside reverse transcriptase inhibitors (NRTIs) are key antiretroviral drugs for the treatment of HIV infection however, as with chemotherapy, the development of sensory neuropathy is a dose-limiting side effect (193). A mouse model of NRTI- induced neuropathy saw a decrease in mRNA of endocannabinoid inactivating enzymes in the paw skin of treated mice compared to controls (194). This may result in lower endocannabinoid deactivating enzyme levels (**Figure 4**) and thus increased levels of endocannabinoids. The endocannabinoid system has been shown to regulate pain transmission in painful peripheral neuropathies (195). Administration of an inhibitor of endocannabinoid hydrolysis resulted in changes in endocannabinoid signalling in paw skin of cisplatin-treated mice (196). These changes correlated with changes in mechanical hyperalgesia (196). As peripheral cannabinoid receptors are largely expressed by immune cells and keratinocytes, these results support a potential interplay between endocannabinoid, immune and peripheral nervous systems in the context of peripheral neuropathic pain states.

### Entrapment and Traumatic Neuropathies

The complex interplay between the cutaneous sensory nervous system and immune system may also contribute to various clinical manifestations of entrapment and traumatic neuropathies. The following section addresses the evidence suggestive of cutaneous neuroimmune interactions in several subtypes of compressive/traumatic neuropathy (carpal tunnel syndrome, trigeminal trophic syndrome and complex regional pain syndrome).

Carpal tunnel syndrome (CTS) is the most common entrapment neuropathy caused by a chronic, focal compression of the median nerve in the carpal tunnel. Patients with CTS experience sensory abnormalities, such as painful paraesthesia, mechanical allodynia, hyperalgesia and loss of thermal sensitivity, which are associated with an altered function of both myelinated and unmyelinated sensory axons (197–199). In addition to direct damage to sensory nerves, CTS is also associated with a systemic inflammatory response in the form of upregulation of serum chemokines such as chemokine (C-C motif) ligand 5 (CCL5), CXCL8, and CXCL10, and growth factors such as vascular endothelial growth factor (VEGF) (200). Despite evidence establishing the role of systemic immune response in CTS-induced neuropathic pain, studies exploring cutaneous neuroimmune interactions in CTS are few. Dermatological signs of CTS commonly quoted in the literature include finger-tip ulceration and recurrent blistering in the dermatomal regions of the medial nerve, hypohidrosis, Raynaud’s phenomenon and irritant contact dermatitis (201–204). Several mechanisms have been proposed to account for these changes such as trauma, infection, and autonomic instability. However, more recently, a new hypothesis involving the formation of immunocompromised districts in the acral areas has been proposed (205). The theory of immunocompromised district refers to a local dysregulation of immune control, which postulates that median nerve compression affects neuropeptide release from peripheral nerve terminals that creates localized and sectorial immune system activation (201). According to this theory, imbalance of immunosuppressive and immunostimulant neuropeptides is likely responsible for the cutaneous manifestations of CTS. A similar mechanism has been proposed for the occurrence of unilateral rosacea secondary to facial nerve palsy (206–208). However, direct evidence for its role in CTS-induced neuropathic pain is lacking.

*Trigeminal trophic syndrome (TTS)* is a rare complication of a trigeminal nerve insult that is characterized by persistent facial ulceration and loss of sensation along the distribution of the affected trigeminal dermatome (209). Facial ulceration occurs most commonly in the nasal ala, cheek and upper lip region and is a result of repeated self-manipulation of the skin in response to facial paraesthesia – involving sensations of burning, tingling, itch or pain in the face (209, 210). TTS can occur because of post-trigeminal ablation, stroke, acoustic neuroma, post encephalitis, vertebrobasilar insufficiency, herpes zoster infection and trauma (209, 211). The pathogenesis of TTS ulceration is poorly understood. There are strong associations between self-provocation of the skin and formation of ulcers (211–213). Other proposed theories include alterations in cutaneous trophic factor secretion (211) and a probable lack of neurotrophic factors such as SP and α-melanocyte-stimulating hormone (212, 213). Indeed, in the case of herpes zoster TTS, mechanisms are likely to involve a reduction in peptidergic fibers in zoster-affected dermatome, thus provoking alterations in neuropeptide release and localized pro-inflammatory response (214, 215). The complexities of neuropeptide dysregulation and its impact on the formation of local immunocompromised districts in TTS are unclear.

*Complex regional pain syndrome (CRPS)* is a devastating complication of minor or moderate tissue injury in the extremities and is characterized by sensory, motor, and autonomic dysfunction (216). In the acute phase, CRPS in an injured limb manifests as severe pain, erythema, swelling and blistering, and sensory disturbances such as allodynia and mechanical and thermal hyperalgesia (216–218). Trophic changes such as altered hair and nail growth can also occur. Chronically, CRPS causes intractable pain that spreads proximally and can affect the contralateral uninjured limb, reduced voluntary muscle control (i.e. weakness, dystonia, tremor and myoclonus) and negative sensory symptoms such as hypoesthesia and hypoalgesia (217–219). The mechanisms of CRPS are complex, involving local inflammatory changes, vasomotor dysfunction, and alterations in the CNS (216, 220). The role of localized inflammation in the context of the skin will be discussed below.

The release of cytokines and growth factors in the skin following tissue injury excites cutaneous nociceptors that induce retrograde depolarisation of small-diameter primary afferents and stimulate the release of neuropeptides such as SP and CGRP, which are found at high levels in CRPS patients (221–224). In CRPS, cutaneous nociceptive pain and neurogenic inflammation are driven by increased extravasation and hampered inactivation of neuropeptides, cytokines and tryptases (225, 226). SP and CGRP have been shown to co-localize with keratinocytes and may even stimulate the expression of cytokines by keratinocytes, which supports their role in mediating neurogenic inflammation in CRPS (216, 227–229). Increase in cutaneous cytokines and tryptases also stimulates proliferation of keratinocytes and mast cell recruitment in the skin (225, 230, 231). Furthermore, studies assessing skin blister fluid and skin biopsies of patients with CRPS have found increased concentrations of pro-inflammatory cytokines TNF-α and IL-6 and a decrease in anti-inflammatory cytokines, which subside with treatment and improvement of symptoms (231–234). Also, serum concentrations of soluble TNF receptor and cytokines TNF-α, IL-1 and IL-8 are increased and anti-inflammatory cytokines IL-4, IL-10 and TGF-β1 are decreased in early CRPS (224, 235). These systemic inflammatory changes do not correlate with disease duration or clinical severity; however, they are associated with mechanical hyperalgesia (235, 236). In contrast, studies have found IENFs to be either reduced (237) or unchanged (238) in CRPS. In chronic CRPS, there is a reduction in local inflammation, with thinning of the skin and less cytokines and macrophages (230). Yet, the mechanisms by which tissue injury stimulates cascading neuroimmune interactions in the skin in CRPS-induced chronic pain are poorly understood.

## Conclusions and Therapeutic Outlook

The skin is a diverse organ protecting the host from the external environment. The skin is home to an array of sensory nerve endings and specialized immune cells, both with heterogeneous functionality. Neuroimmune interplay between sensory nervous system and the immune system in the skin is crucial in homeostasis and disease states. We provide a comprehensive perspective on how peripheral sensory neurons regulate immune cells in the skin and how the cutaneous immune system affects neuronal function, and discuss this bidirectional neuroimmune crosstalk in skin infections, skin disorders and its emerging role in the pathogenesis of peripheral neuropathic pain. While neuroimmune interplay contributes significantly to the pathophysiology of chronic neuropathic pain, other mechanisms such as metabolic disturbances undoubtedly play a pivotal role, and the primary driver of pain chronification remains unknown.

Characterization of the neuroimmune interplay in neuropathic pain states is a highly active field of research with positive potential implications for medical management of some of these conditions. Currently, there are several effective topical agents for the treatment of peripheral neuropathic pain, including lidocaine patches, capsaicin high-concentration (8%) patches, and BTX-A (239–241). While it is known that these drugs affect cutaneous nociceptor nerve endings by blocking sodium channels and increasing ectopic discharge threshold (242), or inhibiting the release of neurotransmitters and neuropeptides (243), their potential effect on cutaneous immune cells remains unclear. An *ex vivo* comparative study found that lidocaine application did not reduce keratinocyte or fibroblast proliferation in skin explants (244). However, a rodent model showed that lidocaine application activated TRPV1 and TRPA1 in the skin and caused TRPV1-dependent release of CGRP from primary afferents (245). Lidocaine-induced release of pro-inflammatory neuropeptides such as CGRP may modulate immune and skin cell function (246). To date, there is minimal evidence of cutaneous neuroimmune regulation by capsaicin 8% patches, although evidence (150) was discussed earlier in this review. A clinical trial investigating BTX-A in the treatment of peripheral neuropathic pain found no changes in concentration of CGRP or SP in skin biopsies of BTX-A-treated patients compared to placebo (247). Targeting specific immune cells, nociceptor terminals and inflammatory mediators in the skin may present novel therapeutic strategies in treating peripheral neuropathic pain.

Given the limited effectiveness of current neuropathic pain treatments, further therapeutic strategies are needed to meet this clinical need. A better understanding of the neuroimmune molecular mechanisms underpinning neuropathic pain pathogenesis may illuminate new avenues of therapeutic research. Given the dysregulation of the cutaneous immune system in neuropathic pain states, one could hypothesize that targeted cutaneous immunomodulatory therapies may present an effective treatment for neuropathic pain, specifically when it is localized. Effectiveness of immunotherapy in treating skin disorders is varied. The successful modulation of pathogenic inflammatory cytokine pathways by targeted immunotherapies to treat psoriasis has shown encouraging results (248). Contrastingly, there is conflicting evidence of efficacy and utility of immunotherapies as a treatment of both AD (249) and ACD (250). Despite the success of preclinical studies, few clinical studies have investigated immune modulators in treatment of neuropathic pain (251). Mixed results and small sample sizes have hampered therapeutic development in this area. Future research into cutaneous targeted immunotherapy to treat neuropathic pain may be fruitful.

Additional research potential may lie in the emerging field of optogenetics. Optogenetics involves the manipulation of cellular activity by introducing and stimulating light-sensitive transmembrane ion channels, commonly in neurons (252). This strategy has been used in mice models to bidirectionally control primary nociceptor function, enabling light-inducible stimulation or inhibition of pain perception (253, 254). This novel technique may allow for precise elucidation of cellular contributors to neuropathic pain and may allow for specific activation or silencing of peripheral nociceptors to modulate neuropathic pain sensation. Notably, a recent study demonstrated that axonal light stimulation of channel rhodopsin-expressing nociceptors resulted in pain behaviors as well as immune reactions in the skin, in the absence of inflammation, by antidromic activation of peripheral terminals (105). Therefore, future studies should examine the effects of such novel therapies on neuroimmune responses in the skin.

## Author Contributions 

GM-T and DL conceived and designed the study. DL drafted the manuscript and prepared the figures. PM contributed to writing sections of the manuscript. All authors contributed to the article and approved the submitted version.

## Funding

This study was supported in part by a grant from the National Health and Medical Research Council of Australia to GM-T (ID # APP1162060). The funder had no role in study design, decision to publish or preparation of the manuscript.

## Conflict of Interest

The authors declare that the research was conducted in the absence of any commercial or financial relationships that could be constructed as a potential conflict of interest.
